# Oscillatory hierarchy in a network of leaky integrate-and-fire neurons with short-term plasticity

**DOI:** 10.1186/1471-2202-14-S1-P5

**Published:** 2013-07-08

**Authors:** Timothee Leleu, Kazuyuki Aihara

**Affiliations:** 1 Institute of Industrial Science, The University of Tokyo, 4-6-1 Komaba, Meguro-ku, Tokyo 153-8505, Japan

## 

**Figure 1:**
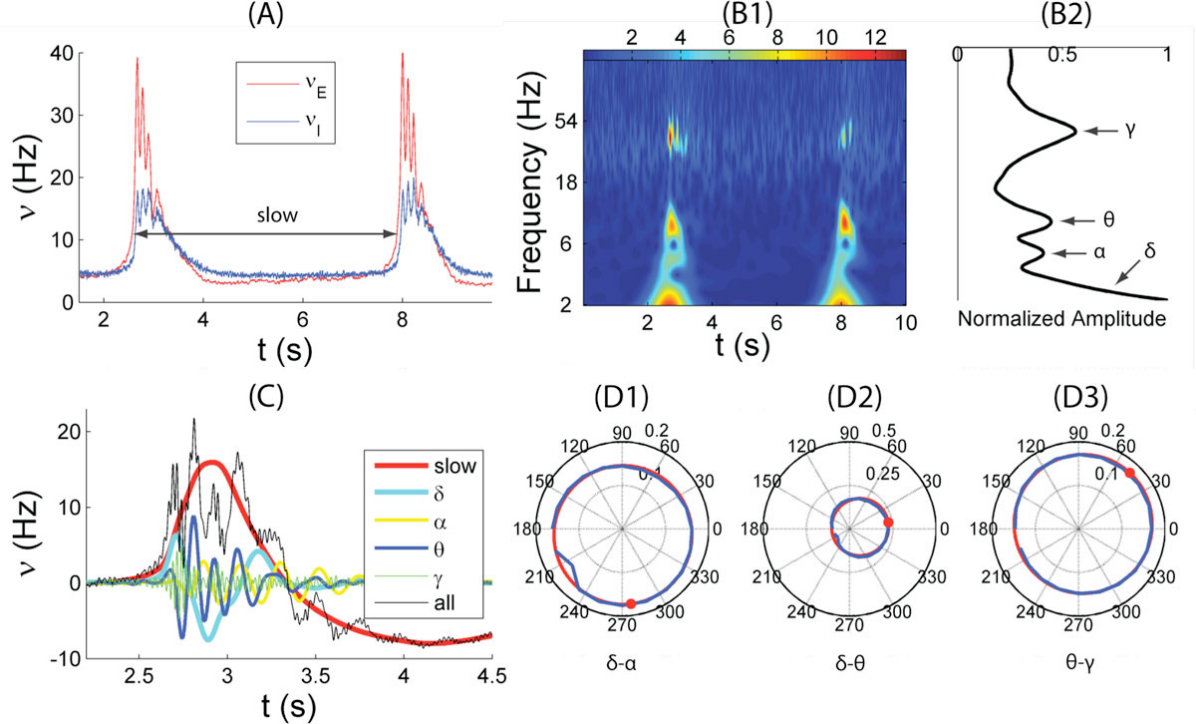
**Simulation in a network composed of 8000 pyramidal cells and 2000 interneurons**. (A) Firing rates of pyramidal cells ν_E _and interneurons ν_I _in red and blue, respectively, smoothen within a window of 25 ms duration. The frequency of slow oscillations is approximately 0.2 Hz. (B1) Continuous wavelet transform of the firing rate ν_I _using Morlet wavelets. The color scale represents the energy for each coefficient of the wavelet decomposition (arbitrary scale). (B2) Normalized cumulated energy for frequencies shown in (B1). (C) Firing rate after applying a band pass filter around frequencies corresponding to slow, δ, α, θ, and γ, respectively. (D1-D3) Cross-frequency coupling between two oscillatory modes f_1 _and f_2 _represented in polar coordinates. Radius and angle are the normalized average amplitude of f_2 _and phase of f_1_, respectively, obtained from the wavelet decomposition (method adapted from [1]). The blue line is the distribution calculated from the simulation of leaky integrate-and-fire neurons. The red line is the fitted von Mises distribution and red dot the phase of f_1 _corresponding to the maximal average amplitude of f_2_. (D1) f_1 _= δ, f_2 _= α. (D2) f_1 _= δ, f_2 _= θ. (D3) f_1 _= θ, f_2 _= γ.

Cross-frequency couplings between oscillatory modes have been observed in cortical and hippocampal local field potentials recorded from the brains of rodents, primates[1] and humans. Multi-unit activity recordings have shown that the highest amplitude of gamma and theta oscillations occur at the rising positive going part of theta and delta oscillations, respectively[1]. We show that a network of leaky integrate-and-fire neurons with short term plasticity between pyramidal cells and interneurons can exhibit a similar oscillatory hierarchy (see Figure 1). Moreover, the network exhibits alpha oscillations which amplitude is modulated by the phase of delta oscillations. There is no phase-amplitude coupling between alpha and theta. Finally, these oscillations are nested in a slower 0.2 Hz oscillation. We study the mechanisms of these oscillatory patterns reminiscent of spontaneous cortical activity[2].
